# *Cryptococcus neoformans* Capsular Enlargement and Cellular Gigantism during *Galleria mellonella* Infection

**DOI:** 10.1371/journal.pone.0024485

**Published:** 2011-09-07

**Authors:** Rocío García-Rodas, Arturo Casadevall, Juan Luís Rodríguez-Tudela, Manuel Cuenca-Estrella, Oscar Zaragoza

**Affiliations:** 1 Mycology Reference Laboratory, National Centre for Microbiology, Instituto de Salud Carlos III, Majadahonda, Madrid, Spain; 2 Department of Microbiology and Immunology and Medicine, Albert Einstein College of Medicine, Bronx, New York, United States of America; Montana State University, United States of America

## Abstract

We have studied infection of *Cryptococcus neoformans* in the non-vertebrate host *Galleria mellonella* with particular interest in the morphological response of the yeast. Inoculation of *C. neoformans* in caterpillars induced a capsule-independent increase in haemocyte density 2 h after infection. *C. neoformans* manifested a significant increase in capsule size after inoculation into the caterpillar. The magnitude of capsule increase depended on the temperature, being more pronounced at 37°C than at 30°C, which correlated with an increased virulence of the fungus and reduced phagocytosis at 37°C. Capsule enlargement impaired phagocytosis by haemocytes. Incubation of the yeast in *G. mellonella* extracts also resulted in capsule enlargement, with the polar lipidic fraction having a prominent role in this effect. During infection, the capsule decreased in permeability. A low proportion of the cells (<5%) recovered from caterpillars measured more than 30 µm and were considered giant cells. Giant cells recovered from mice were able to kill the caterpillars in a manner similar to regular cells obtained from *in vivo* or grown *in vitro*, establishing their capacity to cause disease. Our results indicate that the morphological transitions exhibited by *C. neoformans* in mammals also occur in a non-vertebrate host system. The similarities in morphological transitions observed in different animal hosts and in their triggers are consistent with the hypothesis that the cell body and capsular responses represent an adaptation of environmental survival strategies to pathogenesis.

## Introduction

The yeast *Cryptococcus neoformans* is the causative agent of cryptococcosis, a life-threatening fungal infection. *C. neoformans* infectious particles, probably consisting of spores [1], [2], are believed to be acquired by inhalation. In immunocompetent hosts the infection is often asymptomatic while in patients with impaired immunity, and especially those with advanced HIV infection, there is often extrapulmonary dissemination leading to meningoencephalitis [3], [4]. Although the incidence of HIV-associated cryptococcosis has declined in developed countries with the introduction of the highly active anti-retroviral therapy, its incidence in developing regions, such as sub-Saharan Africa remains very high, causing more than 650.000 deaths per year [5].

*C. neoformans* often causes chronic infection in both humans and animal hosts [3], [6], but this stage of the infection is not well understood. There is indirect evidence that the inability of the host to clear the infection depends on the interaction with phagocytic cells and on the polysaccharide capsule surrounding the cryptococcal cells [7], [8], [9]. In this regard, capsular polysaccharides are potent immunomodulators that interfere with immune responses [10], [11], [12], [13], [14], [15], [16], and consequently the capsule is considered the major virulence factor [17]. However, when *C. neoformans* invades a host it also undergoes changes that contribute to its persistence. These changes include capsule enlargement and the appearance of giant cells [18], [19], [20]. *C. neoformans* can infect a wide variety of hosts, such as the amoeba *Acanthamoeba castellanii*, the nematode *Caenorhabditis elegans*, the slime mold *Dictyostelium discoideum*, insects such as *Drosophila melanogaster* and the Lepidoptera *Galleria mellonella* [21], [22], [23], [24], [25], [26]. Further extending its host range, *C. neoformans* has been shown to infect plants [27].

Some of the alternative models mentioned above are limited by the inability of the host system to survive at 37°C or the difficulty in administering exact fungal inoculum. These limitations do not apply to the *Galleria mellonella* model host. *C. neoformans* can proliferate in the *G. mellonella* haemocoele leading to the eventual death of the caterpillar [23]. Moreover, the *G. mellonella* model has been used to study host defense to fungal pathogens [28].

Although *C. neoformans* morphological changes have been described in yeast isolated from lungs of infected mice [18], [29], their role during infection is not completely understood. The use of an alternative host, such as *G. mellonella*, provides a new system to investigate the importance of specific traits on virulence that has significant advantages over other hosts. We have investigated the interaction between *C. neoformans* and *G. mellonella* and report that cryptococcal infection in this host is also associated with significant morphological changes, such as capsule enlargement and the appearance of giant cells, which play an important role in evading phagocytosis and infection development. Since these processes also occur in vertebrates, our observations indicate similar cellular responses by *C. neoformans* in very different types of hosts.

## Results

### Administration of *C. neoformans* produces a transient increase in haemocyte density

**Figure 1:**
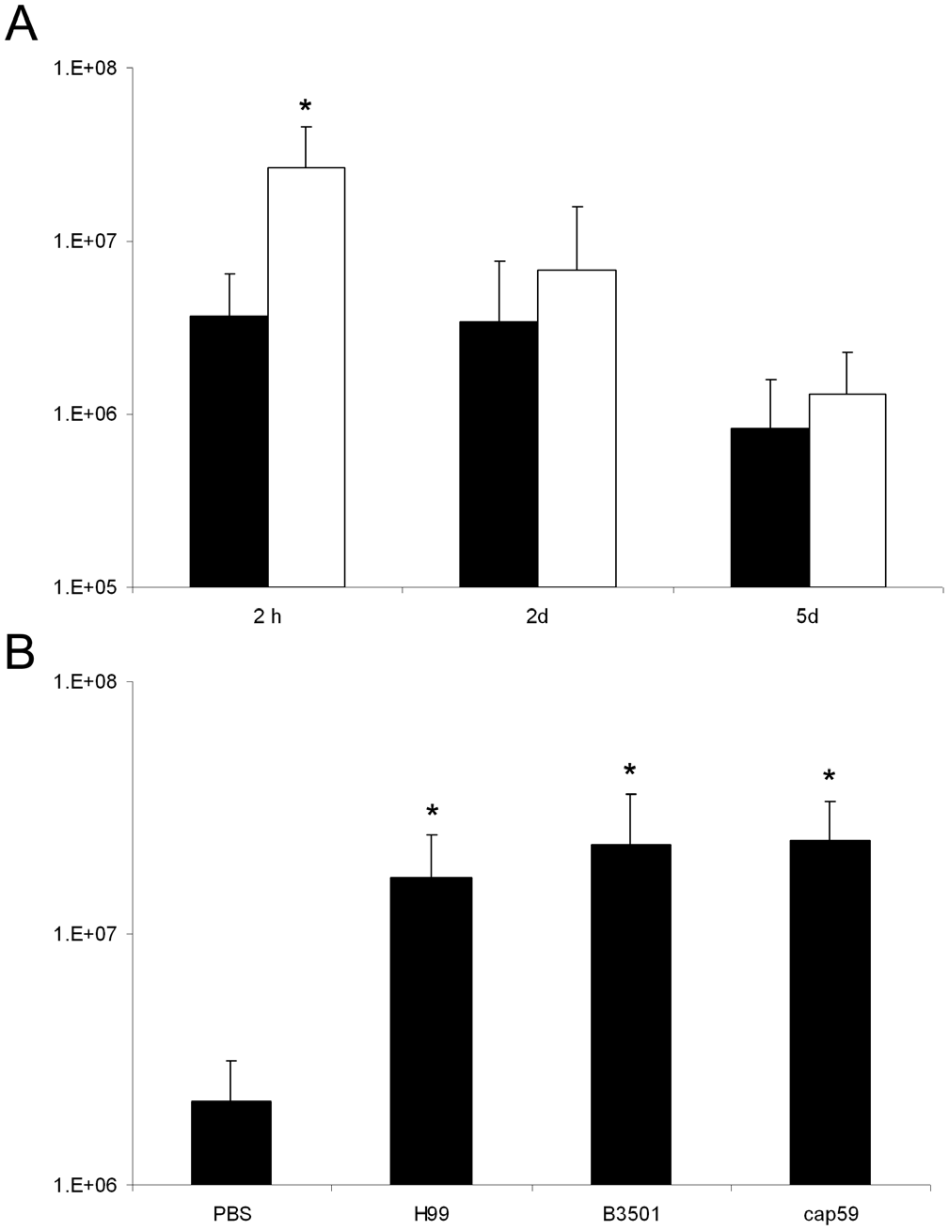
Haemocyte density in larvae after *C. neoformans* injection. **A**) Ten larvae were inoculated with 10^4^ cells from H99 strain and haemocyte densities were assessed at different times (2 h, 2 d and 5 d). Black bars show haemocyte density of larvae injected with PBS and white bars of larvae injected with *C. neoformans*. **B**) Ten larvae per group were inoculated with 10^4^ cells from H99, B3501 and the *cap59* mutant (C536) and haemocyte densities were assessed after 2 h after inoculation as described in Material and Methods. In both A and B, significant differences (*p*<0.05) in haemocyte density relative to that in the PBS treated larvae are indicated with an asterisk. Experiments were repeated 3 times.

To characterize the interaction between *G. mellonella* and *C. neoformans*, we first reproduced the model infection and investigated aspects of the insect immune response during infection with this fungal pathogen. For this purpose, we inoculated larvae with different doses from H99 strain, and observed that the larvae died in a dose dependant manner (data not shown). *Galleria mellonella* induces innate responses, some of them based on the increase in the concentration of haemocytes in the haemolymph at different times [30], [31]. Consequently, we enumerated the number of haemocytes present in the haemolymph at different times, and we observed that larvae injected with 10^4^ *C. neoformans* cells showed a 7-fold increase in the number of haemocytes after 2 h of infection (*p*<0.05). However, this response was not maintained during subsequent days (Figure 1A). Furthermore, we also tested a capsule-deficient strain of *C. neoformans* and its parental strain (*cap59* and B3501, respectively) and we did not observe any statistical differences in the haemocyte density of the larvae infected with these strains (*p* = 0.5) (Figure 1B) indicating that this response is capsule-independent. Heat-killed *C. neoformans* cells were also inoculated in larvae and no increase in haemocyte density was observed (result not shown).

### *C. neoformans* morphogenesis during infection in *G. mellonella*

**Figure 2:**
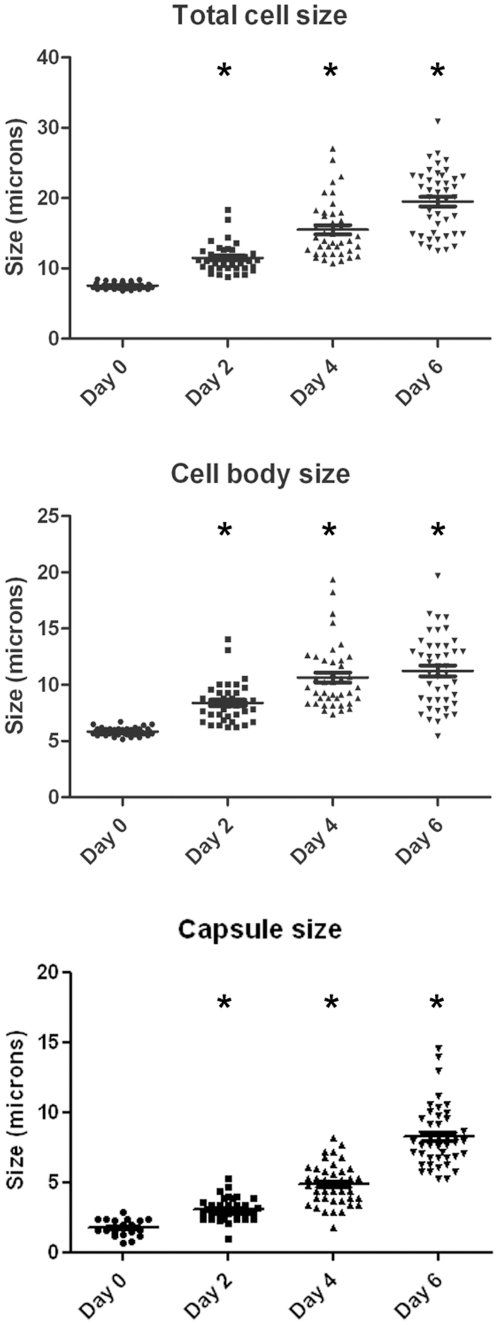
Morphogenesis of *C. neoformans* during infection in *G. mellonella*. Distribution of total cell size, cell body size and capsule diameter of *C. neoformans* cells grown in Sabouraud (T = 0) and recovered from *G. mellonella* caterpillars at different times after inoculation of 10^4^ yeast cells. Total cell size was defined as the diameter of the complete cell including the capsule. The capsule diameter was calculated as the difference between the whole cell size and the cell body size. T-test and scatter plot graphs were obtained using Graph Pad Prism 5. The bar denotes the average of the distribution. Asteriks indicate *p*<0.05.

Since *C. neoformans* manifests morphological transitions during mammalian infection involving changes in the capsule and total size, we investigated whether similar changes occurred during *G. mellonella* infection. Hence, we measured the total cell diameter (capsule included), cell body size (delimited by the cell wall) and capsule diameter of yeast cells recovered from larvae incubated at 37°C at various times of infection. The size of cells grown in Sabouraud overnight used to infect the larvae was considered time zero for infection. *C. neoformans* cells manifested a significant increase in average cell size during infection in *G. mellonella* at all times tested (Figure 2). This increase was due to both an increase in the cell body size and in the size of the capsule when comparing to cells *in vitro* (*p*<0.05, Figure 2).

### Effect of capsule enlargement on phagocytosis

**Figure 3:**
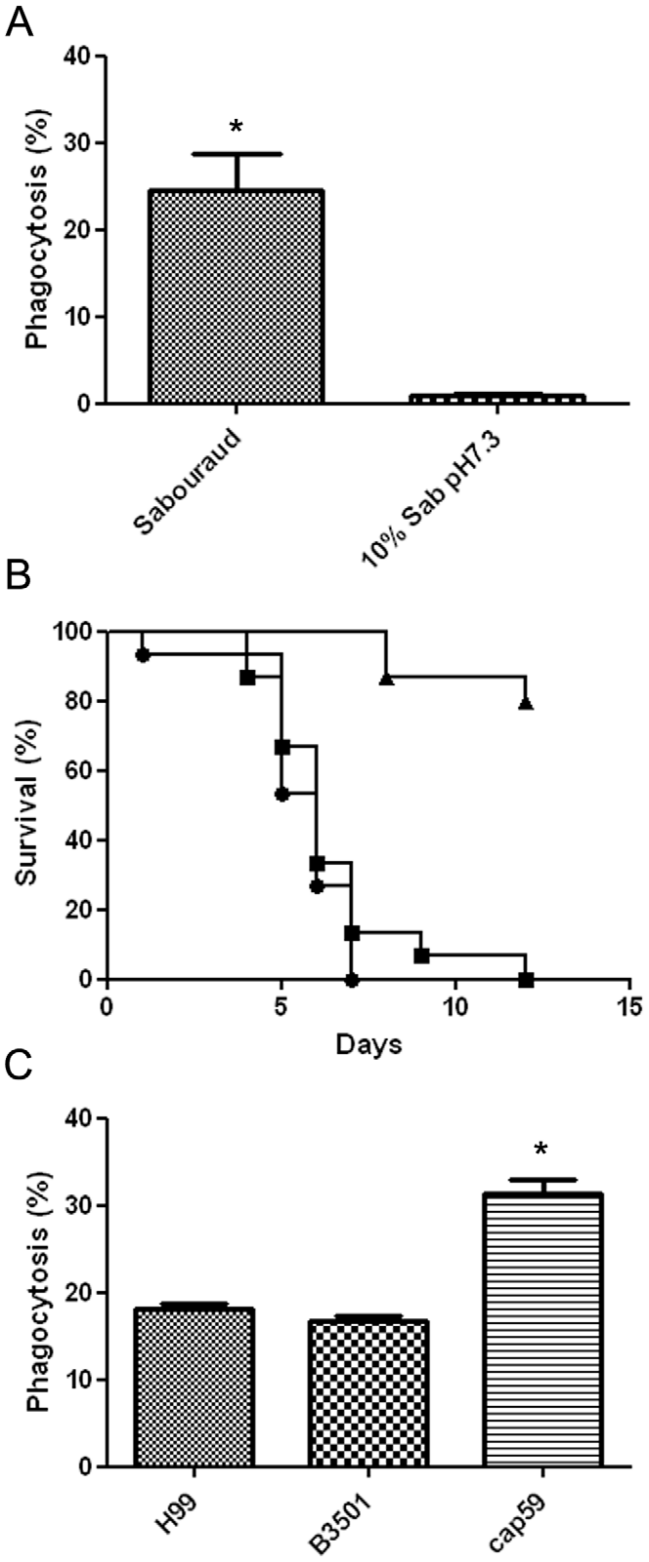
Role of the capsule on phagocytosis by haemocytes. **A**) Cells with small (grown in Sabouraud) and large capsule (incubated in 10% Sabouraud buffered with 50 mM MOPS pH 7.3) were stained with Calcofluor and used to infect caterpillars. Bars show the average phagocytosis at 2 h. *p*<0.05 is indicated with an asterisk. The experiment was repeated twice in triplicates. **B**) Survival curves of *G. mellonella* infected with 10^4^ cells of *C. neoformans* grown in Sabouraud broth and in 10% Sabouraud diluted in MOPS pH 7.3. **C**) *In vivo* phagocytosis using H99, B3501 and *cap59* (C356) strains was performed as described in section A and quantified after 2 h of infection. Bars show phagocytosis average after 2 h. *P* value below 0.05 is indicated with an asterisk.

We then investigated if cryptococcal morphogenesis played a role in virulence in *G. mellonella*. Since initial infection of the larvae was associated with a strong recruitment of haemocytes in the haemolymph, we investigated the importance of capsule growth on haemocyte and phagocytosis. For this purpose, we induced capsule enlargement *in vitro* by incubating the cells in diluted Sabouraud liquid medium at neutral pH (see Material and Methods). When the larvae were infected with cells incubated in Sabouraud broth, we observed that approximately 20% of the haemocytes contained *C. neoformans* cells after 2 h of incubation (Figure 3A). In contrast, capsule enlargement completely inhibited phagocytosis (Figure 3A). However, when we compared the survival of the larvae infected with *C. neoformans* cells with either small or large capsules, we did not observe a difference in virulence (Figure 3B), indicating that initial phagocytosis did not predict the course of infection. To confirm that the capsule had an inhibitory role of phagocytosis by haemocytes, we infected larvae with encapsulated (H99 and B3501) and the *cap59* mutant (C536 strain). Phagocytosis percentage increased to around 30% when using the capsule-deficient strain (Figure 3C, *p*<0.05). No differences in phagocytosis were observed when using encapsulated *C. neoformans* var. *grubii* H99 or *C. neoformans* var. *neoformans* B3501 cells (Figure 3C).

### Temperature dependent morphogenesis of *C. neoformans* in *G. mellonella*

**Figure 4:**
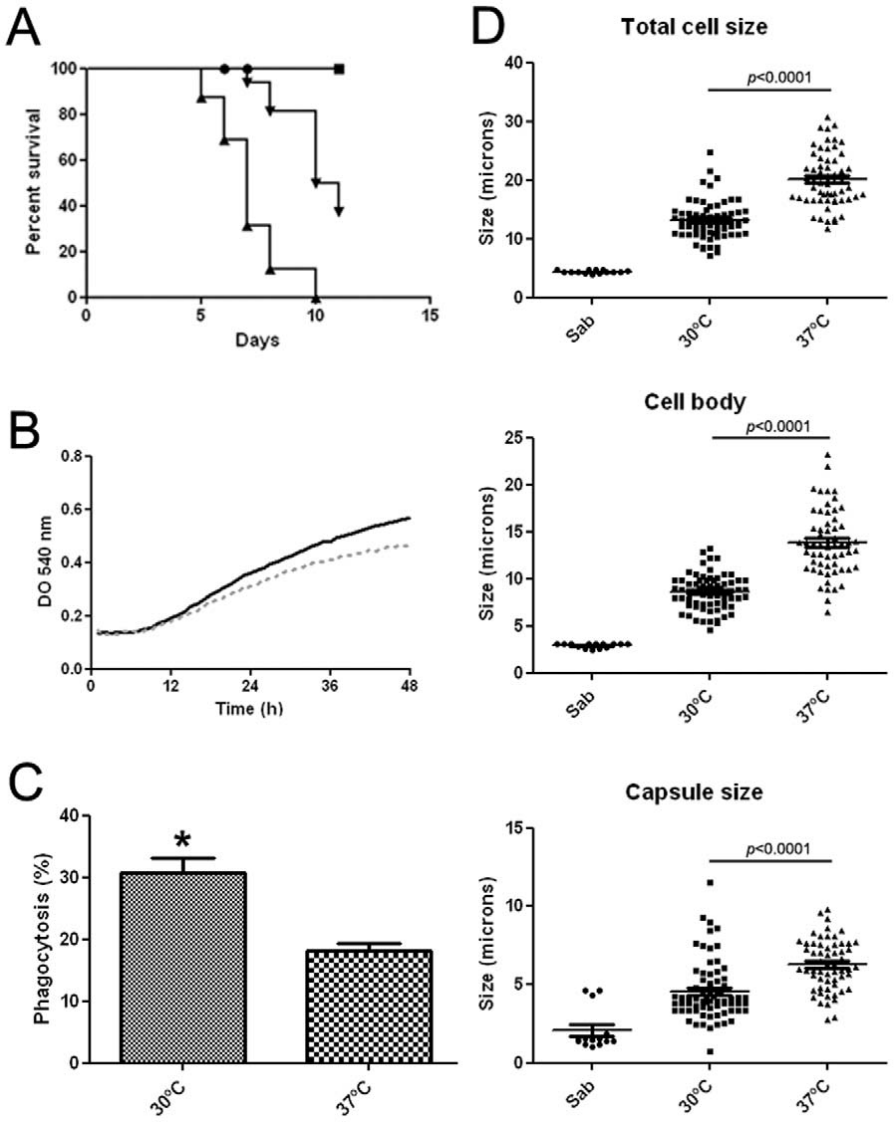
Temperature dependent virulence and morphogenesis of *C. neoformans* in *G. mellonella*. **A**) Survival curves of *G. mellonella* infected with 10^4^ H99 cells. Sixteen larvae per group were incubated at 30°C (▾) and 37°C (▴). A group of larvae were injected with PBS and kept at 30°C (•) and at 37°C (▪) to control the trauma of the injection. **B**) Growth curves of H99 strain at 30°C (Black line) and at 37°C (grey dotted line) during 48 h. **C**) *In vivo* phagocytosis was performed as described in Material and Methods and quantified after 2 h of incubation of the larvae at 30°C and 37°C. *P*<0.05 is indicated with an asterisk. **D**) Distribution of total cell size, cell body size and capsule diameter of *C. neoformans* cells grown in Sabouraud (Sab) and recovered from *G. mellonella* caterpillars at different temperatures after 6 d of infection. T-test and scatter plot graphs were performed using Graph Pad Prism 5. The line in each sample denotes the average of the distribution.

The virulence of *C. neoformans* in *G. mellonella* was reported to be decreased at 30°C compared to physiological mammalian temperature [23]. Consequently, we investigated whether this difference in virulence correlated with differences in morphogenesis. We first reproduced the temperature-dependent virulence phenotype, and as shown in Figure 4A, we confirmed that virulence of *C. neoformans* was enhanced at 37°C. We wanted to rule out that the enhanced virulence at 37°C was due to a better growth of the yeast at this temperature, so we performed growth curves at 30 and 37°C. We found that the growth rate was higher at 30°C than at 37°C (Figure 4B). In addition, we studied whether phagocytosis of *C. neoformans* was affected by the incubation temperature. To this end, we performed *in vivo* phagocytosis assays at both temperatures (Figure 4C). Two groups of larvae were inoculated with 10^6^ cells of *C. neoformans* previously stained with Calcofluor White and incubated at both temperatures. After 2 h, haemolymph was obtained from 3 different larvae in every group to quantify phagocytosis. We observed that phagocytosis occurred more efficiently at 30°C than at 37°C (*p* = 0.0011). This data suggests that the immune system of the larvae is impaired at 37°C. However, and as shown in Figure 3, the initial phagocytosis does not correlate with the outcome of the infection, so we investigated the magnitude of morphological changes at 30 and 37°C. *C. neoformans* exhibited a significant increase in the size of both the cell body and the capsule during infection in *G. mellonella* at both temperatures (Figure 4D). However, this increase was statistically higher in cells from larvae at 37°C than from those kept at 30°C (*p*<0.05) (Figure 4D).

### Capsular rearrangements during infection in *G. mellonella*

**Figure 5:**
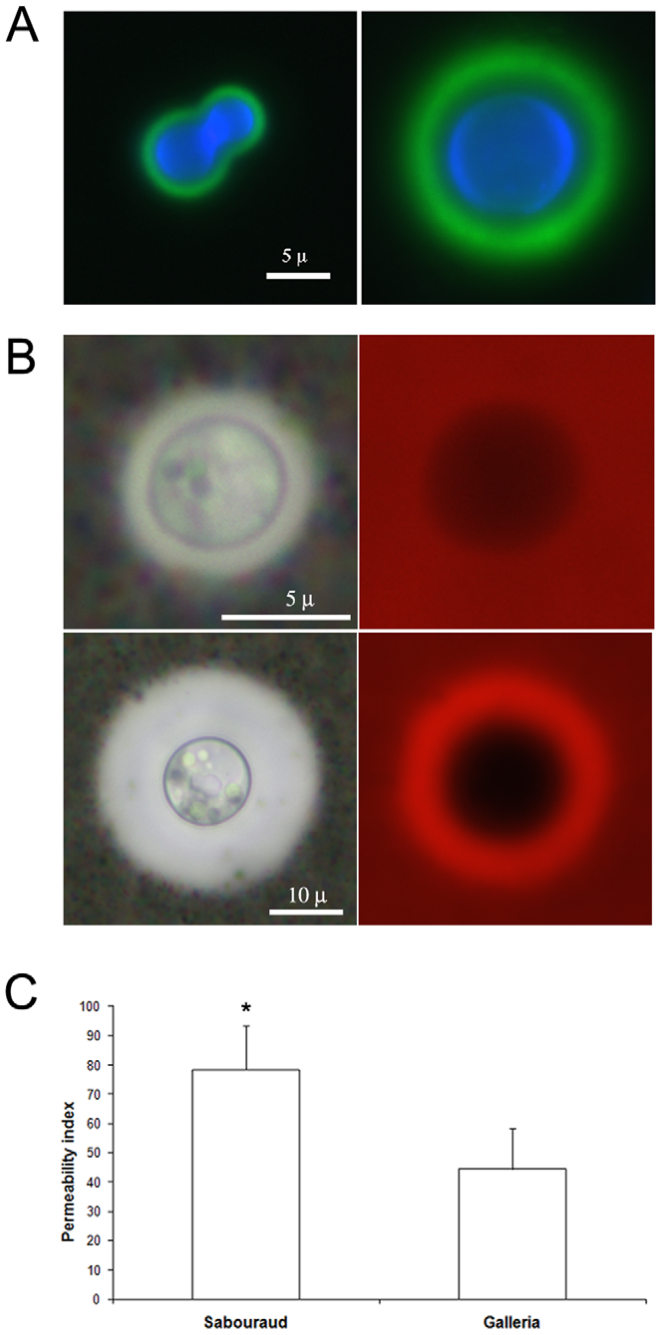
Capsular permeability of *C. neoformans* cells isolated from *G. mellonella*. **A**) Immunofluorescence staining of the capsule with mAb18B7-Alexa 488 (green fluorescence) and of the cell body (blue fluorescence) of cryptococcal cells grown in Sabouraud broth and recovered from caterpillars infected with 10^4^ cells per larva after 6 d. Scale bar in left panel applies to both. **B**) Penetration of 70 KDa dextran Rhodamine labeled into the capsule and estimation of total capsule size by India ink staining. Upper panel shows cells grown in Sabouraud broth and lower panel cryptococcal cells recovered after 6 d from caterpillars infected with 10^4^ cells per larva. Scale bar in left panel applies to both. **C**) Distribution of permeability index calculated for cells grown in Sabouraud liquid medium (Sabouraud) and for cells recovered from infected larva (Galleria). T-test *p* value = 0.0006.

We investigated if during *G. mellonella* infection, the capsule underwent changes not only in size, but also in structure. So we stained the capsule with mAb 18B7 conjugated to Alexa-488 and compared the immunofluorescence pattern of cells grown *in vitro* and cells recovered from caterpillars. In all cases, we found a clear annular binding pattern of the Ab to the yeast cells (Figure 5A). However, for cells recovered from *G. mellonella*, the mAb bound primarily to the outer regions of the capsule and there was little or no fluorescence signal from the regions close to the cell wall (Figure 5A), which suggested that infection in *G. mellonella* was associated with an increase in capsule density that reduced penetrability to the inner capsule. To confirm this idea, we investigated the penetration of 70 kDa fluorescent dextrans into the capsule. In cells grown *in vitro*, the dextran penetrated in the capsule to regions close to the cell wall, yielding a penetration to approximately 75% of the capsule diameter (Figure 5B, 5C). However, in cells isolated from *G. mellonella*, the dextran could not penetrate to the regions close to the cell wall (Figure 5B,C; penetration index around 45%), implying a reduction in permeability consistent with an increase in capsule density during infection in the insect.

### Capsular enlargement *in vitro*

**Figure 6:**
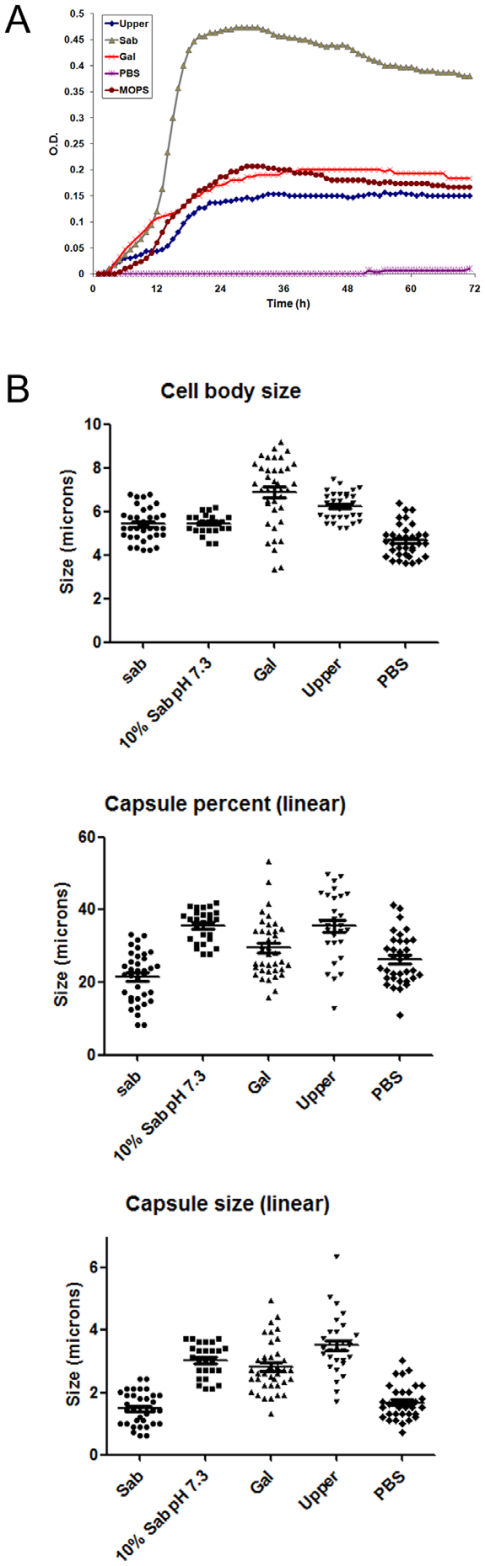
*In vitro* capsule and cellular enlargement of *C. neoformans* in *G. mellonella* extracts. Larvae were homogenized and different extracts were prepared as described in Material and Methods. **A**) Growth curves in Sabouraud (▴), 10% Sabouraud diluted in 50 mM MOPS pH 7.3 (•), total *G. mellonella* extract (**x**), Upper phase of *G. mellonella* extract, containing polar lipids (♦) and PBS (*) at 37°C during 3 d. **B**) Distribution of total cell size, cell body size and capsular diameter of *C. neoformans* cells in Sabouraud (Sab), Sabouraud buffered with MOPS (10%Sab pH 7.3), *G. mellonella* extract (Gal), upper phase of *G. mellonella* lipid extraction (Upper phase) and PBS at 37°C for 10 d. T-test comparing cells in different media to cells grown in Sabouraud broth and scatter plot graphs were done using Graph Pad Prism 5. The line in each sample denotes the average of the distribution. *P*<0.05 is indicated with an asterisk.

In addition to the *in vivo* experiments, we also performed *in vitro* experiments to study if *C. neoformans* induced capsule growth in the presence of specific caterpillar factors. In addition to a whole caterpillar extract, we also investigated a polar lipidic fraction, since similar fractions obtained from *Acanthamoeba castellanii* and murine macrophages can induce capsule growth [32]. Sabouraud liquid medium, PBS and 10% Sabouraud in 50 mM MOPS buffer pH 7.3 were also tested. We first analysed the growth of *C. neoformans* in each of the different fractions. Growth curves during the first 72 h revealed very similar *C. neoformans* growth in diluted Sabouraud medium (pH 7.3), complete *G. mellonella* extract and in the polar lipid rich fraction (Figure 6A). No growth was observed in PBS (Figure 6A). When we analyzed the size of the cells after 10 d, we observed that incubation of *C. neoformans* in complete *G. mellonella* extract and in the polar lipid extract resulted in cell size enlargement, due to both cell body and capsule increase, compared to the same cells incubated in Sabouraud broth (Figure 6B) or in PBS in which cells showed a reduced size. In addition, the increase in capsule percentage obtained when *C. neoformans* was incubated in the polar fraction was similar to that observed in the capsule inducing medium (10% Sabouraud at neutral pH, see Figure 6B).

### *C. neoformans* forms giant cells during infection

**Figure 7:**
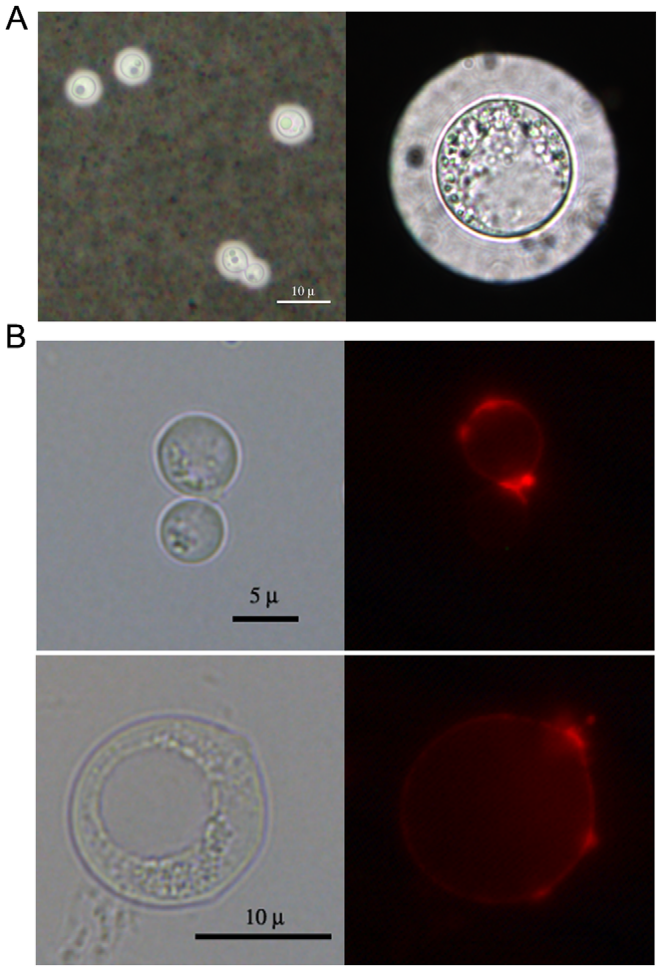
*Cryptococcus neoformans* forms giant cells in *Galleria mellonella*. **A**) Figure shows photographs of *C. neoformans* cells suspended in India ink after overnight growth in Sabouraud broth and giant cells recovered from *G. mellonella* after 6 d of infection. Scale bar applies to both panels. **B**) Binding of wheat germ agglutinin to *C. neoformans* as described in Material and Methods. Upper panels show cells grown in Sabouraud browth and lower panels show a representative cell recovered from caterpillars after 6 d of infection.

In addition to capsule enlargement, another significant morphological change associated with cryptococcal infection in murine models is the appearance of giant cells (above 30 µm) [19], [20]. In our *G. mellonella* model less than 5% of the cells recovered from day 6 infected larvae reached over 30 µm and thus could be considered giant cells (Figure 7A). However, cells with a diameter between 20–30 µm could be observed after 4 days in larvae infected with 10^4^ yeast cells. This phenomenon was also observed in caterpillars infected with 10^2^ and 10^3^ inocula, but only after 6 days of infection (data not shown). We observed that giant cell formation involved a 75-fold increase in cellular volume relative to cells grown in Sabouraud liquid medium. The giant size of these cells was achieved not only by capsule enlargement, but also by an increase of the cell body size (Figure 7A).

Chitin-like structures in the capsule were reported by the specific binding of fluorescent wheat germ agglutinin, which binds to sialic acids and β-1,4-N-acetylglucosamine (GlcNAc) oligomers [33]. We used WGA to ascertain whether these structures were also present in giant cells recovered from *G. mellonella*. Cells grown *in vitro* bound WGA especially at the neck between the mother cell and the bud (Figure 7B). In cells recovered from caterpillars, these structures were particularly prominent (Figure 7B).

### Giant cells virulence in *G. mellonella* model host

**Figure 8:**
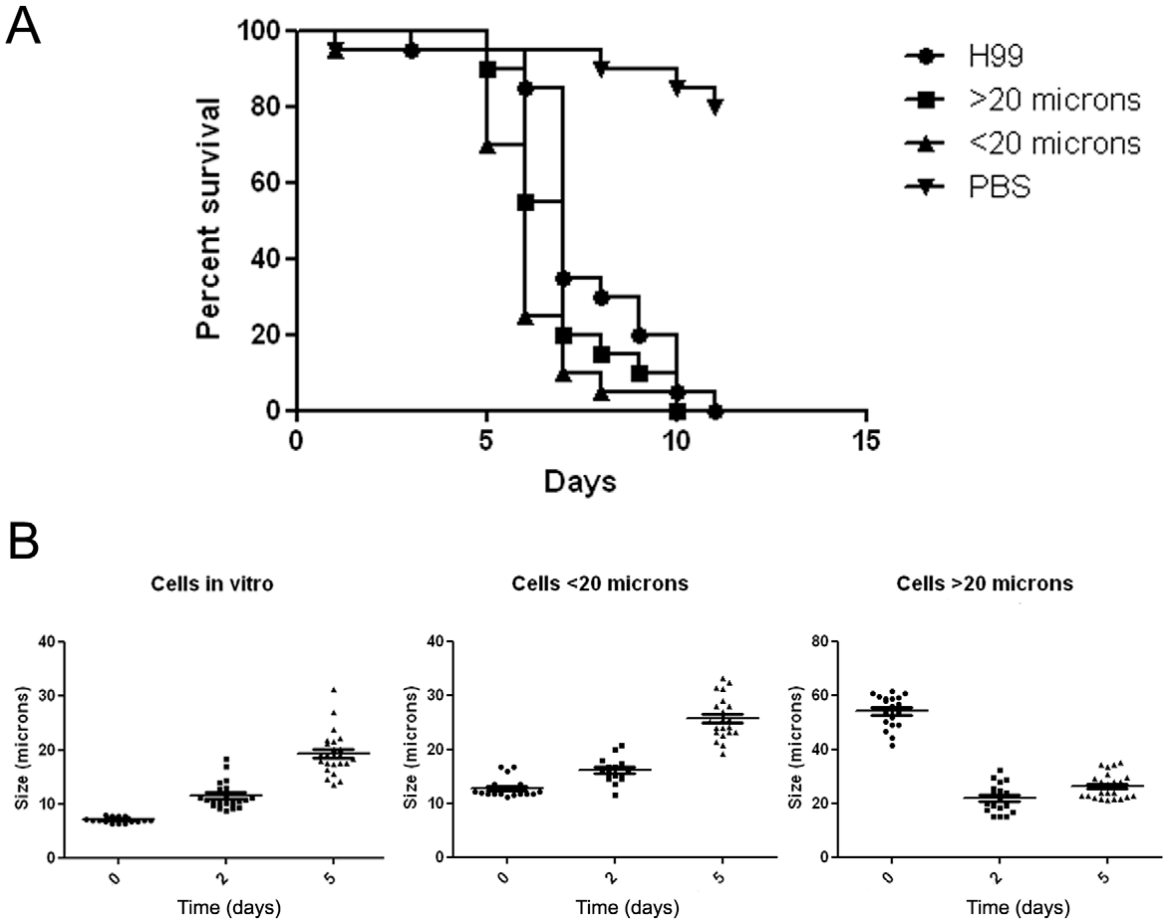
Survival of *G. mellonella* infected with giant cells isolated from mice. **A**) Balb/c mice were infected with H99 strain, and after 17 d, yeast cells recovered from the lungs were separated according to their size. Two different samples were obtained: cells smaller than 20 µm or cells larger than this size. Yeast cells incubated overnight in Sabouraud were carried out in parallel. Twenty larvae per group were inoculated with 10 µL of 3×10^5^/mL suspension of the 3 different *C. neoformans* preparation: Sabouraud (indicated in the figure as H99), cells from mice of regular size (<20 µm) and giant cells (>20 µm). Since yeast cells from mice were treated with Streptomycin to reduce bacterial contamination (see Material and Methods), a group of larvae was inoculated with 1% Streptomycin in PBS as a control. **B**) Distribution of cells recovered from caterpillars from the different groups at different times of infection (days 0, 2 and 5).

We used the *G. mellonella* model to investigate the virulence of giant cells formed in mice. For this purpose, we isolated giant cells from lungs of 3 week-infected mice. To separate giant from regular cells in the lung extract, we filtered the yeast suspension through 20 µm pore size filters, which have been shown to retain most of the giant cells [20]. As a control, we also used the cells that passed through the filter (of which the majority were of regular size) and cells grown in Sabouraud liquid medium overnight. We found that giant cells had the same virulence capacity as cells of regular size obtained from *in vitro* or *in vivo* (Figure 8A). To investigate whether the size of the giant cells was maintained during infection, fungal cells were recovered from larvae from every group at different times and their sizes measured. An increase in total cell size (capsule included) was found in cells recovered from larvae infected with cells grown *in vitro* and in those infected with *C. neoformans* with a cell size below 20 µm isolated from mouse lungs (Figure 8B). In contrast, in larvae infected with giant cells, the cells recovered after 2 d from the caterpillars had a decreased size, and giant cells were hardly found (Figure 8B). Nevertheless, after 5 d of infection cells isolated from larvae seemed to begin to increase their size again and they showed an average size of 26 µm (Figure 8B).

## Discussion

Virulence of human pathogens has classically been studied in mammalian models, with mice being the most widely used species. In recent years there has been increasing interest in the use of other model hosts, given concerns about the expense and bioethical issues involved in vertebrate animal experimentation. However, the use of these alternative models must be validated before the results obtained can be extrapolated to more complex models, such as the mouse. In this sense, it is important to establish whether the interaction between non-vertebrate model hosts and pathogens results in similar phenomena as observed with more conventional types of hosts. *Galleria mellonella* caterpillars have been used for the study of pathogenesis with such different pathogens as *Pseudomonas aeruginosa*, *Proteus mirabilis* and *Escherichia coli* [34], [35], [36], [37], [38]. Among human fungal pathogens, *Candida albicans* and some *Aspergillus spp*, such as *A. fumigatus* and *A. flavus*, also kill caterpillars [28], [31], [39], [40]. The immune response elicited by *G. mellonella* during *C. neoformans* infection is very different from that elicited in mammalian cells. As invertebrates, insects lack adaptive immune responses and do not produce immunoglobulins. However, insects have powerful innate immune mechanisms that include the presence of haemocytes with phagocytic activity [41], [42], [43], [44], which in some aspects resemble the activity of macrophages and neutrophils from the innate immune system of mammalian organisms. In addition, another part of the innate immune response in insects depends on the role of reactions catalyzed by a phenoloxidase that enable to encase pathogens in melanin [45]. *C. neoformans* induced a 7-fold increase in haemocyte density in caterpillars during the first 2 h of infection. This acute response was capsule and strain independent and not maintained in time, but required living cells. Hence, *C. neoformans* in mammals and insects is likely to encounter different challenges for survival. Consequently, the question of how *C. neoformans* responds in the setting of mammalian and insect infection is relevant for our understanding of pathogenesis and the origin of virulence of this fungus.

Morphological changes have been described in mouse models during the infection in the lungs. These morphological changes include capsule enlargement and the formation of giant cells, which could be important for the persistence of the yeasts in the host. We have confirmed that similar changes occur during infection in *G. mellonella*, which supports the idea that this model can be used to study morphological aspects of *C. neoformans* infection, which might be then extrapolated to mouse models. In addition, *G. mellonella* offered a simple model to study the importance of morphogenesis in virulence. In this sense, we found that capsule enlargement inhibited phagocytosis. The mechanism by which capsule growth impairs phagocytosis by haemocytes is unknown, but a similar situation is observed with complement-mediated phagocytosis of *C. neoformans* by murine macrophages [46]. But it cannot be discarded that capsule growth inhibits phagocytosis by producing cells of larger size which cannot be ingested by haemocytes. However, the initial size of the capsule did not influence virulence of the strain, most probably due to the fact that during infection, cells with small capsule manifest capsular enlargement, and consequently, become similar to those incubated in capsule inducing medium. Moreover, our findings suggest that the initial phagocytosis does not correlate with the subsequent development of the disease, so the consequences of the increase in haemocyte density and the exact role of phagocytosis in protection against cryptococcal infections deserve future study. In agreement with the antiphagocytic role of the capsule, we observed that phagocytosis was increased when we used a capsule-deficient strain, a situation with strong resemblance to the observation that acapsular strains are readily ingested by mammalian macrophages [47], [48]. The capsule completely inhibits phagocytosis, and in the case of mammalian macrophages, phagocytosis of regular encapsulated *C. neoformans* strains completely depends on the presence of opsonins, such as complement or antibodies (see review in [9]), and for this reason, few hours after intratracheal infection, most of the fungal burden is found inside phagocytic cells [49]. In *G. mellonella*, we found that around 20% of haemocytes contained *C. neoformans* cells after 2 h of infection, and we believe that this phagocytosis is also produced by insect opsonins.

We noted another instance where capsule and cell enlargement correlated with virulence, which is the different virulence shown by *C. neoformans* at different temperatures [23]. This difference was not explained by a better growth of the yeast at high temperature, but correlated with decreased phagocytosis and a larger capsule, which we believe translates into phagocytic avoidance. This data is also in agreement with previous findings that demonstrated that capsule enlargement *in vitro* was enhanced at physiological temperature [50]. In addition to this enhanced fungal response, the immunity of the larvae seems to be diminished at 37°C, as shown by the reduced phagocytosis measured at this temperature.

Capsular enlargement in *G. mellonella* was associated with a decrease in the permeability of the capsule, a phenomenon that might have also important consequences for the recognition of the pathogen by the host. Increases in capsule density can affect penetration of antifungal molecules and the binding of opsonins on the capsule. Capsular rearrangement in the *C. neoformans* capsule was described both during infection [51], [52] and *in vitro* [50], [53], [54], and this phenomenon has been associated with cellular aging [53]. Our findings highlight that this particular ability of *C. neoformans* to modulate capsular structural features might have important consequences for multiple types of interactions, with both the environment and the host.

The molecular mechanisms that allow capsule enlargement are unknown. A common feature of capsule growth is that it occurs in poor nutritional conditions, where normally the growth rate of the yeast is impaired, so it is possible that one parameter that contributes to capsule growth is a decrease in cell division rate. Although this concept might be true, we believe that capsule enlargement is also induced by host specific factors. We were able to induce capsule enlargement by incubating cryptococcal cells with caterpillar extracts, and in particular, with the polar lipidic fraction, which is in agreement with recent findings of *C. neoformans* using extracts from *Acanthamoeba castellanii* and macrophages [32]. In these conditions, growth rate was compromised, so we argue that decreased growth in required to induce capsule enlargement in the presence of specific inducers, such as host lipids. Conservation of this effect in insects supports the view that phospholipid sensing might be a general response that is stimulated by common elements present in different hosts.

The formation of *C. neoformans* giant cells could pose a major problem to the immune system simply by virtue of their size [19], [20]. In this work, we isolated giant cells from caterpillars after 6 d of infection at 37°C. Furthermore, in the *G. mellonella* model, cells of 20–30 µm could be observed by 4 d post-infection in larvae infected with 10^4^ inoculum, and at day 6 in larvae infected with 10^2^ and 10^3^ cells. Consequently, we surmise that infection with 10^2^ or 10^3^ was better controlled by caterpillar haemocytes. Giant cells isolated from *G. mellonella* manifested several similarities with the giant cells isolated from mice, such as the presence of chitin-like structures which might be involved in the movement of the bud through the capsule of the mother cell [33]. However, we also noticed structural differences between giant cells obtained from *G. mellonella* and mice. Giant cells from mice were consistently larger than those obtained from the insect, and had prominently enlarged cell walls [20]. Although it is possible that giant cells formed in insects and in mammals are different, we believe that the most likely explanation for the differences observed is the time of infection. In mice, we routinely recover the giant cells after 3 weeks of infection, so the yeasts have more time to become larger and develop specific structural features than in the insect model, since they are obtained only after 6 d of infection due to short lifespan of infected caterpillars. This makes very difficult to obtain giant cells from infected insects for a longer time. In addition, giant cells from mice were polyploid [19], [20], which suggests a mechanism of giant cell formation by endoreduplication. In the case of giant cells obtained from *G. mellonella*, this aspect could not be studied due to the relatively scarce number of giant cells recovered from the larvae, which precluded the use of specific techniques, such as DNA staining and analysis by flow cytometry, to measure the DNA content. Despite these differences, our results indicate that *G. mellonella* offers a simple system to study the early stages of giant cell formation and could be used as a rapid and inexpensive model to screen for mutants affected in this process.

In addition, *G. mellonella* can be used to study specific aspects of cryptococcal virulence. For example, we observed that giant cells isolated from mice can kill the caterpillars in a very similar way to cells of regular size, obtained from both *in vivo* and *in vitro*. Furthermore, 2 d after infection with giant cells, most of the cells recovered from larvae were not giants, indicating that giant cells *in vivo* produced smaller cells, as we observed *in vitro* [20]. Since giant cells have been shown to be more resistant to some stress factors [20], the observation that giant cells can produce infection provides support for the notion that such cells could contribute to latency and reactivation by virtue of their relatively invulnerability to host defense mechanisms, since they are too large to be ingested [19], [20]. In this regard, we hypothesize that giant cells could linger in the lung and provide a source of infection with the daughter cells being capable of extrapulmonary dissemination, when hosts become immunosuppressed.

*C. neoformans* factors involved in mammalian pathogenesis are also involved in the survival of *C. neoformans* within non mammalian hosts [23], [55], [56]. In this work, we have shown that *G. mellonella* provides a useful model to study morphological cellular and capsular changes previously detected in mammal host. Moreover, the ability to recover giant cells from caterpillars could significantly enhance our ability to study this enigmatic cell type using *G. mellonella* as it constitutes an inexpensive non-vertebrate animal host. Our results also contribute to the understanding of the origin of cryptococcal virulence since they imply the existence of similar survival strategies in mammalian and insect hosts despite major differences in the immune response of these animals. Consequently, these observations support the notion that the pathogenic potential of *C. neoformans* for animal hosts is a consequence of selection pressures in the environment that resulted in attributes also suitable for animal virulence [26], [57].

## Materials and Methods

### Strains and media

The *Cryptococcus neoformans* var. *grubii* H99 strain, also known as ATCC 20882 [58], [59], was used throughout the study. *C. neoformans* var. *neoformans* B5301 strain [60] and the capsule-deficient strain C536 (which has a deletion in the *CAP59* gene, [61]) were also used in some experiments. *C. neoformans* cultures were grown in liquid Sabouraud medium at 30°C with moderate shaking (150 r.p.m). To induce capsule enlargement, the cells were incubated in 10% Sabouraud buffered at pH 7.3 with 50 mM MOPS [62].

### *Galleria mellonella* survival experiments

The *Galleria mellonella* larvae (Vanderhorst Wholesale, Inc, St Marys, OH and Mous Livebait R.J., The Netherlands) used for experiments were selected to be similar in size and absent of any grey markings for reproducible results.

The selected larvae were inoculated with 10 µL of a yeast suspension prepared at different cell densities by an injection in the last left pro-leg using a 26 gauge needle with Hamilton syringes. The syringes were prepared prior to the experiment by cleaning them with bleach and ethanol. Before injection, the pro-leg area was cleaned with 70% ethanol using a swab. After injection, caterpillars were incubated in 90 mm plastic Petri dishes (Soria genlab, S.A., Madrid, Spain) at 37°C or at 30°C, and the number of dead caterpillars was scored daily. A group of caterpillars was inoculated with PBS in each experiment to monitor killing due to physical injury and another group of caterpillars without any manipulation was carried out in parallel as an untreated control. Killing curves were plotted and estimation of differences in survival was analyzed by the Kaplan-Meier method using Graph Pad Prism 5 (La Jolla, CA, USA). Every experiment was repeated at least twice, obtaining always similar results. Data presented here are from a representative experiment.

### Isolation of *C. neoformans* cells from *G. mellonella*

To isolate the yeasts from *G. mellonella*, larvae were smashed using cell strainers of 100 µm pore size (BD Falcon, Erembodegren, Belgium) and 10 mL syringe plungers (BD Plastipak, Madrid, Spain). Homogenates were collected in 1 mL of PBS and samples were washed twice and suspended in 150 µL of PBS.

Fungal cells were suspended in India ink (Remel Bactidrop, Lenexa, KS, USA), observed by microscopy and photographed. Photographs were taken using a Leica DMI 3000B microscope in all experimental work. In parallel, cryptococcal cells grown overnight in Sabouraud broth were observed by microscopy, photographed and used to infect the larvae.

Cell and capsule sizes were measured using Adobe Photoshop 7.0 (San Jose, CA). Total cell size was defined as the diameter of the complete cell including the capsule. Capsule size was calculated as the difference between the diameter of the total cell and the cell body diameter, defined by the cell wall. T-test and scatter plot graphs were done using Graph Pad Prism 5 (La Jolla, CA, USA). A *P* value<0.05 was considered significant.

### *G. mellonella* extract preparation and lipid extraction

Twenty to thirty larvae were smashed in PBS and the homogenate was collected and centrifuged at 3000 r.p.m. for 5 min. Total extracts were obtained after filtering the supernatant through 0.22 µm pore size filters (Millex, Co. Cork, Ireland). To obtain polar lipidic fractions, we followed the protocol described in [32]. Briefly, the total extract was incubated with a mixture of chloroform and methanol (2∶1, v∶v) for 3 h on a benchtop rocker at room temperature. The samples were then centrifuged for 10 min at 2500 r.p.m for phase partitioning. The upper phase was collected and dried overnight in a vacuum centrifuge. The pellet was finally suspended in PBS.

### Capsular enlargement assay upon fractionation

Total *G. mellonella* extract and the lipidic fraction were tested for capsule enlargement with *C. neoformans* strain H99. Sabouraud liquid medium, diluted Sabouraud (10%) in 50 mM MOPS pH 7.3, and PBS were also tested in parallel. A ninety six-well plate was used to incubate 10^4^ cells per well in triplicates with the different media at 37°C with shaking for 10 d. Capsular size was measured as described above.

### Immunofluorescent staining of the capsule

Suspensions of *C. neoformans* recovered from caterpillars after 6 d of infection and *C. neoformans* grown in Sabouraud broth overnight at 30°C were stained with mAb18B7 [63] conjugated to Alexa 488 at a final concentration of 10 µg/mL for 30 min at 37°C in the dark. Samples were washed 3 times with PBS and incubated with 0.1% Uvitex for 15 min at room temperature in dark. Samples were then washed 4 times and the pellets were suspended in 0.1 M n-propyl-gallate (Sigma, St. Louis, MO, USA) in PBS. Photographs were taken using an Olympus IX70 microscope (Olympus America, Melville, NY).

### Wheat germ agglutinin staining

To evaluate the presence of chitin-like structures, fungal cells grown in Sabouraud liquid medium at 30°C overnight and cells recovered from *G. mellonella* larvae 6 d post-infection were stained with wheat germ agglutinin (WGA) as reported previously [33]. Briefly, the cells were washed with PBS, suspended in 4% p-formaldehyde cacodylate buffer (0.1 M, pH 7.2) and incubated for 30 min at room temperature. The fixed cells were then washed with PBS and suspended in 100 µL of 5 µg/mL WGA conjugated to Alexa 594 (Molecular Probes, Invitrogen) for 1 h at 37°C. Cell suspensions were mounted on glass slides and photographed with a Leica DMI 3000B fluorescence microscope.

### Capsule penetrability assay

Capsule penetrability assays were performed as described in [52] to define the porosity of the capsule to macromolecules. Briefly, suspensions of *C. neoformans* recovered from caterpillars 6 d after infection and *C. neoformans* grown in Sabouraud broth overnight at 30°C were suspended in a mixture of India ink and 3.6 µg/mL of 70 KDa Rhodamine fluorescent dextrans (Sigma). Samples were taken from those suspensions and photographs were taken in bright field and in Rhodamine filter. Total cell size and cell body size were measured in India ink suspensions as described above. The area of the capsule where the dextran did not penetrate was also measured by Rhodamine. The penetrability index was calculated as follows: Total capsule size was estimated after suspension in India ink, and the penetration of Rhodamine labeled dextran was measured in parallel by subtracting the diameter not penetrated by the fluorescent dextran from the total cell body. The permeability index was defined with these two values as the percentage of the capsule that was penetrated by the fluorescent dextran referred to the total size of the capsule. Cells were measured using Adobe Photoshop 7.0 (San Jose, CA) and statistics and graphs were obtained using Graph Pad Prism 5 as explained previously.

### *C. neoformans* growing curves in Sabouraud liquid medium at 30°C and 37°C

*C. neoformans* strain H99 was grown overnight at 37°C with moderate shaking. Suspensions of *C. neoformans* at 10^5^/mL and 10^3^/mL were prepared in Sabouraud and 200 µL were inoculated in a 96-well plate (Costar, NY, USA) and incubated for 48 h with moderate shaking every 30 min in an iEMS Spectrophotometer (Thermofisher). Optical density at 540 nm was measured every hour and graphs were plotted using Graph Pad Prism 5. The growth curves were performed at both 30°C and at 37°C in different days in triplicates.

### *In vivo* phagocytosis assays

*C. neoformans* was grown in liquid Sabouraud medium as described above and stained with Calcofluor White (Sigma Aldrich, St. Louis, Mo, USA) at 10 µg/mL for 30 min at 37°C. In some experiments, yeast cells with enlarged capsules induced by an overnight incubation in 10% Sabouraud buffered at pH 7.3 with 50 mM MOPS were used. After incubation, cells were washed twice with PBS and suspended at 10^8^ cells/mL. Larvae were infected with 10 µL of the inoculum (10^6^ cells) and incubated at 37°C. In some experiments, a group of infected larvae was incubated also at 30°C. After 2 h, haemolymph was obtained from the caterpillars by insertion of a lancet into the haemocoele. Haemolymph was collected in eppendorf tubes containing an equal amount of an anticoagulant and antimelanization buffer IPS (Insect Physiological Saline:150 mM sodium chloride, 5 mM potassium chloride, 10 mM Tris-HCl pH 6.9, 10 mM EDTA and 30 mM sodium citrate) and were centrifuged at 1500 r.p.m for 5 min to separate the haemocytes from the humoral fraction. Pelleted haemocytes were washed twice with the same buffer and the haemocytes were placed on coverslips and allowed to adhere for 40 min. Coverslips were placed on the slides with Fluoromount G and pictures were taken to quantify the phagocytosis with a Leica DMI 3000B fluorescent microscope. Experiments were performed in different days in triplicates. T-test (Microsoft Excel) was used to analyze the data and a *p* value<0.05 was considered significant.

### Determination of haemocyte density

Larvae were inoculated with 10 µL of a 10^6^/mL suspension of either H99, B3501, C536 strains. In some experiments, *C. neoformans* cells killed by incubation at 60°C for 45 min were also inoculated as control. Haemocyte density was assessed by obtaining the haemolymph from 10 larvae after different times (2 h, 2 d, 5 d) after the administration of the inoculum as described above. Haemolymph was immediately diluted 1∶1 in IPS buffer to prevent melanization. Density was assessed by counting the haemocytes using a haemocytometer. All determinations were performed on 4 independent experiments.

### *Galleria mellonella* survival experiment with cells recovered from infected mice

BALB/c mice were infected intratracheally with 50 µL of a 2×10^6^/mL suspension in PBS of H99 cells grown overnight at 30°C in Sabouraud liquid medium as described in [64]. Autoclaved food, bedding and bottles were used to maintain the animals. To minimize bacterial contamination, 1 g/L tetracycline hydrochloride (Sigma Aldrich, St. Louis, MO, USA) was added to the sterile drinking water. After 17 d, mice were euthanized using CO_2_ and lungs were isolated. All the animal procedures used in this study were approved by the Bioethical Committee for Animal Welfare for the Instituto de Salud Carlos III (approved protocol PA-39). Lung tissue was then homogenized in 10 mL of PBS containing 1% Streptomycin and 1 mg/mL collagenase (Roche, Mannheim, Germany). The use of Streptomycin avoided bacterial contamination through the purification procedure, and for that reason, was included in all the buffers and solutions used. The cell suspension was incubated for 90 min at 37°C with occasional vortex agitation, and washed several times with sterile distilled water. Cell suspension was filtered through a 20 µm filter (Millipore, Ireland) to separate cells with a cell body diameter larger than 20 µm from smaller cells. In parallel, cells from H99 strain incubated in Sabouraud broth overnight at 30°C were also examined as control. After determining the cell density of the different cell fractions recovered from the mice, suspensions of 3×10^5^ cells/mL in PBS containing 1% Streptomycin were prepared. An aliquot of these samples was plated on Sabouraud agar plates to confirm the number the CFUs and the absence of bacterial contamination of the samples.

Twenty larvae per group were injected as described previously. A group of larvae was injected with PBS containing 1% Streptomycin. Larvae were incubated at 37°C and the number of dead caterpillars was scored daily. Differences in the survival of the various groups were analyzed by the Kaplan-Meier method using Graph Pad Prism 5 (La Jolla, CA, USA).

At selected time intervals after inoculation larvae were homogenated and cryptococcal cells were recovered from every group. Cells were suspended in India ink and several pictures were taken. Total cell size, was measured using Adobe Photoshop and graphs and statistic analysis were performed using Graph Pad Prism 5 (La Jolla, CA, USA).

### Statistical analysis

Graphs and statistical comparisons (Student's *t*-test) were performed using Graph Pad Prism 5 (La Jolla, CA, USA) and Microsoft Excel.
